# The Experience of Sexual Stigma and the Increased Risk of Attempted Suicide in Young Brazilian People from Low Socioeconomic Group

**DOI:** 10.3389/fpsyg.2017.00192

**Published:** 2017-02-22

**Authors:** Angelo Brandelli Costa, Andrew Pasley, Wagner de Lara Machado, Ernesto Alvarado, Luciana Dutra-Thomé, Silvia Helena Koller

**Affiliations:** ^1^Research Group Prejudice, Vulnerability and Psychosocial Processes, Department of Psychology, Pontifícia Universidade Católica do Rio Grande do SulPorto Alegre, Brazil; ^2^Department of Education and Social Work, University of AucklandAuckland, New Zealand; ^3^Research Group Psychological Assessment on Human Potential, Department of Psychology, Pontifícia Universidade Católica de CampinasCampinas, Brazil; ^4^Center of Psychological Studies, Institute of Psychology, Universidade Federal do Rio Grande do SulPorto Alegre, Brazil

**Keywords:** suicide, sexual stigma, young people, low-SES, minority stress, network analysis, Brazil

## Abstract

This study was intended to analyze the intersection of experience of sexual stigma low-socioeconomic status, and suicide attempt amongst young Brazilians (11–24 years old). In each of the data collection periods (2004–2006: *n* = 7185; 2010–2012: *n* = 2734), participants completed a questionnaire-based instrument. Network analysis provided support for a Minority Stress Model, oriented around whether participants had experienced sexual stigma. Although suicide attempts decreased by 20% for participants who had not experienced sexual stigma, there was a 60% increase for those who had experienced sexual stigma. Of particular note were the increases in rates of reported community and familial physical assault, molestation, and rape for those who had experienced sexual stigma. An analysis of centrality statistics demonstrated that both experiences of this Minority Stress Model were fundamentally different, and that those disparities increased over the time frame observed in this study. At the center of this model, shortest paths statistics exhibited a direct conditioned connection between experiencing sexual stigma and suicide attempts. We discuss the social and historical contexts that contributed to these dynamics, and emphasize the need for policy change.

## Introduction

Almost universally, normative sexuality has been limited to heterosexual frameworks, which is to say that social infrastructures are, explicitly or implicitly, designed to privilege the lives of those who embody this standard, and disadvantage those who do not (Warner, 1993). Sexual stigma refers broadly to “all facets of stigma associated with same-sex desires, sexual behaviors, and relationships, as well as sexual minority communities” (Herek, 2016, p. 397). In line with Herek's definition 2016, sexual stigma can manifest itself institutionally, indirectly affecting particular individuals and as a felt stigma or perceived stigma, in terms of physical and psychological violence. Non-heterosexuals (or those perceived to be non-heterosexual) are often subject to a slew of prejudice and discrimination such as bullying (Poteat and Espelage, 2005), physical abuse (Saewyc et al., 2004), sexual abuse (Gold and Marx, 2007), and violence (Gruenewald, 2012). Those who had experienced sexual stigma also tend to have significantly worse educational level (Kosciw et al., 2013), earning (Blandford, 2003), employment (Pizer et al., 2011), physical (Hatzenbuehler et al., 2014a,b) and mental health outcomes (King et al., 2008). The internalization of sexual stigma among sexual minorities also manifests itself as a self-stigma: “a self-directed prejudice, whereby the self-concept is congruent with the stigmatizing responses of society” (Herek, 2016, p. 398). Of particular concern, sexual stigma have been shown to increase the risk of developing depression (Hatzenbuehler et al., 2008) and suicide risk (Plöderl et al., 2014). Recent meta-analytic research has demonstrated that non-heterosexual individuals have an increased risk for suicide (Haas et al., 2010; Plöderl et al., 2013). These disparities are further augmented in contexts with limited or non-existent state protection (Hatzenbuehler et al., 2009). In 2014, the World Health Organization issued a report aimed at increasing awareness and suicide prevention in the global public health agenda, with a specific emphasis on the vulnerability of non-heterosexual cohorts (WHO, 2014).

Despite a 29.5% increase in Brazilian suicide rates between 1980 and 2006, the national rate is still considered low (.49 per 10,000; Lovisi et al., 2009), relative to worldwide suicide rates (11.6 per 10,000; Värnik, 2012). More alarmingly, suicide attempt rates in Brazil have been estimated at 3.1% (Bertolote et al., 2005; *n* = 516). In another study, focused on a cohort from 1980 to 2000, Mello-Santos et al. (2005) found that the greatest increase in suicide rates was in the 15–24 age range (190%). Furthermore, Souza et al. (2002) demonstrated the distribution of suicide mortality among young people (15–24 years old) across 11 Brazilian state capitals. All cities analyzed exhibited increased suicide mortality (average increase of all 11 cities.35 to.50 per 10,000 inhabitants, 1979–1998).

A 2016 Brazilian report on sexual and gender minority violations provided additional evidence that analyses of young people are a vital focus, as 61.2% (*n* = 1695) of cases of abuse committed against participants in their sample were experienced by those between the age of 15 and 29 (Secretaria de Direitos Humanos, 2016). Nonetheless, there is still no literature in Brazil showing the health impact of this kind of violation. Bostwick et al. (2013) validated our concern, as they found that experiencing sexual discrimination put individuals at risk of significant mental health disorders. Additionally, research by Mustanski and Liu (2013), and Stone et al. (2014) has found a high risk of suicide amongst non-heterosexuals aged 15–24. In an attempt to understand why minority populations demonstrate significantly reduced well-being, Meyer (2003) conceptualized the Minority Stress Model. This model posits that health outcomes are products of the interactions between coping resources and stressors (Meyer et al., 2008). Stressors are experienced as direct discrimination; expected discrimination; and internalized prejudice toward one's (perceived) identity (Meyer, 1995). Conversely, a sense of belonging to a supportive community (McLaren et al., 2008), positive educational experiences (Russell et al., 2001), religiosity (Kralovec et al., 2014), and high positive self-esteem (Corning, 2002) are associated with a reduced vulnerability for mental health morbidity. Dunn et al. (2014) provided one example of a sexual minority stress model in Brazil, demonstrating that the effect of concealment of sexual identity on depressive symptomatology was contingent on the level of resilience. Our research was intended to replicate important aspects and extend on these studies in order to analyze the intersection of sexual stigma and suicide attempts amongst young Brazilians.

In addition to the factors described above, those who live in poverty are more vulnerable to a wide range of stressful life circumstances, from financial uncertainty (Milne and Plourde, 2006) and food insecurity (Olson, 1999; Drewnowski and Specter, 2004) to homelessness (Ziesemer et al., 1994), and institutionalization (Hutz and Silva, 2003). However, they are not a homogenous group. Concerning socioeconomic status, Patel and Kleinman (2003) performed a literature review, which demonstrated that poverty, as a whole, had only a weak direct relation to higher rates of psychological dysfunction. However, variables that tend to occur at higher rates in low-SES contexts—the experience of financial, food, or residential insecurity, rapid social change, exposure to violence, and risk of poor physical health—each had stronger associations with mental health vulnerabilities. Rasmussen et al. (1997) and Díaz et al. (2001) found that negative mental health outcomes were related to experiences of sexual discrimination and poverty. Rasmussen et al.'s (1997) study is particularly pertinent, as it focused on young people. Recognizing the high degree of variation in conditions between various social strata, we sought to concentrate on groups that have been identified as particularly vulnerable to minority stress conditions. Due to the level of need in these populations, our study was designed to focus on low-SES young people.

Consequently, this study sought to investigate the extent to which sexual stigma experiences affected the prevalence and dynamics around suicide attempts in two cross-sections of low-SES Brazilian young people. Implementing network analysis (Epskamp et al., 2012), we sought to visualize the systems of protector and stressor variables, and determine whether a sexual minority stress model existed in these contexts. Measures of minority stress included experience of prejudice (including heterosexism, racism, and sexism), community and familial abuse, and negative life events. Extant literature on suicidality supports the inclusion of measures of positive self-esteem (Johnson et al., 2010), educational fulfillment (Lewis et al., 1988; Kalafat and Elias, 1995; Portzky and van Heeringen, 2006), and community support (De Man and Labrèche-Gauthier, 1991), as buffers against stressors. Additional demographic measures included gender, ethnicity (skin color; see Williams et al., 1997, for an account of the role of skin color in Brazil), and religion (Stack and Lester, 1991).

Whilst we are utilizing a non-*a priori* network analysis, we do expect a separation between stressor and protective variables to emerge from the data, and a clear relationship between sexual discrimination and suicide attempts to become apparent. Whilst we believe that there is sufficient evidence to suggest that there will be a positive association between sexual stigma experience and suicide attempt, shortest path and centrality statistics will allow us to clarify whether this is a direct or mediated relationship; how strong that relationship is; and the extent to which the model revolves around that relationship. Subsequently, we hypothesized that there will be fundamental differences between the configuration and associations that emerge for sexual stigma experiences of young people.

## Methods

### Procedures

#### The overall project

The Brazilian Youth Research survey (Koller et al., 2005; Liborio and Koller, 2009; Dell'Aglio and Koller, 2011) was designed to investigate risk and protective factors for young Brazilians in a national sample of low-sociodemographic neighborhoods. The first cross-section was collected between 2004 and 2006, whilst the second was collected between 2010 and 2012.

Sampling procedures were informed by census data (IBGE, 2001), using neighborhood sociodemographic indicators to select low income samples in each of the five regions of Brazil: Porto Alegre and Rio Grande, in the south; Recife and Fortaleza, in the northeast; Campo Grande, Brasilia, Goiania and Hidrolandia, in the central west; Manaus and Belem, in the north; and São Paulo, Vitória, Belo Horizonte, Presidente Prudente, Arcos, Serra, Vila Velha, Cariacica, and Viana, in the southeast.

Five basic indicators were chosen to analyze the status of each of the neighborhoods: income and literacy level of household head, household construction material (masonry, wood, etc.), and the existence of piped water and sewerage systems. They are appropriate criteria within the Brazilian context, since they mark differences between lower and higher income neighborhoods. The indicators “household construction material” is particularly relevant in Brazil to identify low-income contexts (ex.: slums), where usually houses are built with non-durable material (e.g., wood; straw); as well as the inexistence of piped water and sewerage systems. The neighborhoods that were below a cut line for at least two indicators were included in the potential sample size. Thereafter, a stratified sample of the geographic regions of the city was performed. From that subset, 10 neighborhoods were chosen from each of the cities, based on those criteria. Public schools were randomly chosen from the subsets. Thereafter, all students in the selected schools were invited to participate in the study. This process was repeated for the second data collection.

The samples for each cross-section are independent (i.e., no repeated measures), The length of each collection period can be attributed to the duration of the process. In both cross-sections, information was collected using an interview-based questionnaire specifically developed for this study, which included: biosociodemographic information; familial composition; education; employment; health; quality of life; sexuality; risk behaviors (drug use, suicide attempts, sexual risk behaviors, and violence or abuse); stressors (family violence, disease, disability, discrimination, conflict with the law, institutionalization, poverty, separation/loss in the family, familial suicide); protective factors (leisure, support network, communal cohesion, familial satisfaction, friendships); and personal variables (spirituality, moral values, self-esteem, optimism, meaning in life, humor, altruism/sociability, self-efficacy, future outlook).

The database was composed of data collected by surveys that were approved by the Ethics Committees of the universities of the participating sites. The ethical aspects that guarantee the integrity of the participants were ensured, based on the Brazilian legislation that is in accordance with international ethical parameters for research involving human subjects. School coordination signed a Term of Agreement concerning the involvement in the research and parents or guardians signed a term that allowed the adolescents to participate in the study. Adolescents over 18 signed the term by themselves. There was no financial compensation to partake. The instrument was applied collectively in classrooms in accord to institutional time schedules in paper version, with the presence of the research team to supervise and clarify their doubts concerning the questionnaire items.

#### The current study

The results were analyzed with the postulated minority stress systems as the predictor variable, and suicide attempt as the dependent variable. From the overall survey, we extracted the measures found in both studies and examined how they operated with respect to suicide attempts, focusing on the role of having experienced sexual stigma. This experience was identified using the item “I have been discriminated because of my sexual orientation,” as sexual identity is a less reliable indicator of stressful experience.

Protective factors included measures of positive self-esteem, educational satisfaction, and community support. Risk variables constituted measures of abuse within the participant's community and family, prejudice, and negative self-esteem. Biosociodemographic information was used to infer how these variables operated across gender, skin color, and belief in God. Race/skin-color/ethnicity was designated using the Brazilian Institute of Geography and Statistics census categories: white, black, yellow (mostly East Asians), and indigenous. One other category, *pardo*, was used, which commonly refers to Brazilians of a mixed-race, typically a mixture of white, Afro and native Brazilian. The category “indigenous” does not reflect a color, but a race. A preliminary analysis of ethnic categories found no significant difference in the relationship between separate racial categories and suicide attempt, but we sought to include white/young people of color discrepancies to determine whether whiteness, as a privileged category, provided a buffer against minority stress. Thus, for this study, participants were divided in two groups: white and young people of color.

### Participants

The first cross-section was made up of 7,185 young Brazilians. The second was comprised of 2,734 young Brazilians, bringing the total to 9,919 participants over the entire study. The average age of the participants was 16.15 years [95% CI (16.12, 16.19 years); SD 1.9 years; Mdn 16 years], and the range of the participants' ages was 11–24 year. Demographic characteristics can be found in Table 1.

**Table 1:** **Demographic characteristics**.

	**Total**		**2004–2006**		**2010–2012**	
	***n***	**(%)**	***n***	**(%)**	***n***	**(%)**
**GENDER (*N* = 9863)**						
Women	5492	55.68	3937	54.86	1555	57.87
Men	4371	44.32	3239	45.14	1132	42.13
**PLACE OF RESIDENCE (*N* = 9919)**						
State capital	8109	81.75	5821	81.02	2288	83.69
Other municipality	1810	18.25	1364	18.98	446	16.31
**REGION (*N* = 9919)**						
Southeast	3265	32.92	2941	40.93	324	11.85
Northeast	2303	23.22	1265	17.61	1038	37.97
Center West	1823	18.38	1776	24.72	47	1.72
South	1720	17.34	937	13.04	783	28.64
North	808	8.15	266	3.70	542	19.82
**RACE/SKIN-COLOR/ETHNICITY (*N* = 9731)**						
Colored	6098	61.48	4244	60.43	1854	68.46
White	3633	36.63	2779	39.57	854	31.54
**BELIEF IN GOD (*N* = 9294)**						
Yes	9050	97.47	6466	98.21	2593	95.68
No	235	2.53	118	1.79	117	4.32
**SEXUAL STIGMA (*N* = 9341)**						
No experience of sexual stigma	8665	92.76	6296	93.18	2369	91.68
Experience sexual stigma	676	7.24	461	6.82	215	8.32

### Network analysis

To determine the existence of a Sexual Minority Stress Model, we administered Network Analysis (Schmittmann et al., 2013), a machine learning technique that allowed us to visualize patterns that emerge from the data (i.e., non-a priori models). Network analysis utilizes a range of algorithms to produce various spatially meaningful graphical representations of data, illustrating the pairwise interactions (edges) that constitute the systems of variables (vertices). The Fruchterman-Reingold algorithm enables the data to be portrayed in relative space, wherein variables with stronger associations hang together, while less strongly related variables are repelled from one another (Fruchterman and Reingold, 1991). To begin with, we produced association networks using bivariate correlations, using qgraph R package (Epskamp et al., 2012). Bivariate correlations networks are useful to visualize general patterns in data, but also can bring lot of spurious relationships, making these networks to be fully connected. Further algorithms can be used to discriminate key variables in the whole system, enabling the control of spuriousness.

Next, we sought to hone our explanatory precision by executing the partial correlation network. These networks are a class of model were edges represent conditional (i.e., partial) association between variables. This model is known as Pairwise Markov Random Fields, and is estimated through L1-regularized neighborhood regression. The regularization is achieved by a least absolute shrinkage and selection operator (LASSO; Friedman et al., 2008) which controls the model sparsity. After several iterations, extended Bayesian information criteria (EBIC, Chen and Chen, 2008) is applied for model selection for a level of penalty parameter (in general between.25 and.5). As qgraph is designed for dichotomous, ordered categorical or continuous data, biosociodemographic were necessarily represented on two-levels (e.g., representing skin color on a continuous scale is meaningless). Most of the others variables in the model were dichotomised to increase its sensibility, for example, zero representing a complete absence of a variable (e.g., has not experienced punches or slaps), whilst a one represented some degree of a variable (i.e., any degree of punches or slaps). For this reason, we used non-parametric approximations of correlation matrix (Liu et al., 2012) and fitted a series of Gaussian graphical models (GGM, Lauritzen, 1996), including overall, 2004–2006, 2010–2012 cross-sections. Network analysis allowed us to detect structural differences between stressor and protective factors surrounding suicide attempts amongst low-SES young Brazilians. In particular, it enabled us to observe whether equivalent models existed for participants that have experienced sexual stigma (SS) vs. those that have not (NSS), and between each cross-section. The variables used in the present study are described in Table 2.

**Table 2:** **Detailed legend for Figures 1–3, and Tables 3, 4**.

**Abbreviation**	**Meaning in the network**
Gen	Gender
Sch1	Feels good in their school
Sch2	Enjoys going to school
Sch3	Likes most of their teachers
Sch4	Wants to continue at the same school
Sch5	Has confidence in teachers and others members of the school
Sch6	Has confidence in school friends
Aggr1	Has received a scolding
Aggr2	Has received punches and slaps
Aggr3	Has been assaulted with an object
Aggr4	Has had their body touched without consent
Aggr5	Has (been the victim of) forced sexual relations
Com1	Has suffered a large scolding in their community
Com2	Has suffered punches and slaps in their community
Com3	Has suffered assault with an object in their community
Com4	Has had their body touched without consent in their community
Com5	Has suffered forced sexual relations in their community
Prej1	Has suffered prejudice in the place their lives
Prej2	Has suffered prejudice as a result of their sex
Prej3	Has suffered prejudice as a result of their sexual orientation
Prej4	Has suffered prejudice as a result of their skin color
Prej5	Has suffered prejudice as a result of studying in public school
Prej6	Has suffered prejudice as a result of their parents occupation
Prej7	Has suffered prejudice as a result of socioeconomic level
Prej8	Has suffered prejudice as a result of their religion
Prej9	Has suffered prejudice as a result of physical appearance
Prej10	Has suffered prejudice as a result of having disabilities
NLE1	Their socioeconomic status dropped
NLE2	One or more of their relatives have been unemployed
NLE3	Their parents are separated
NLE4	Has already been institutionalized
NLE5	Has run away from home
NLE6	Has lived on the street
NLE7	Has slept on the street
NLE8	One or more of their relatives has been arrested
NLE9	Has suffered an accident that led to disability
NLE10	Someone important to them has died
NLE11	Has starved
NLE12	Has been arrested
NLE13	Has had problems with the justice system
CPF1	Feel like they belong to the community
CPF2	Feel that most people are trustworthy
CPF3	Feels safe in their community
CPF4	Has support from their community
CPF5	Have support from community institutions
CPF6	Their community has improved
PSS1	Feel that they are a person of value
NSS1	Feel ashamed to be the way they are
NSS2	Feel like they “can't do anything good”
PSS2	Feel that they are capable of doing as much as others
NSS3	Feel they are a failure
NSS4	Feel that they are useless
PSS3	Believe they have good qualities
PSS4	Feel they have reasons to be proud of themselves
PSS5	Satisfied with themselves
PSS6	Positive attitude toward themselves
Rel	Believe in God
SAT	Has attempted suicide
SO	Sexual Stigma
SC	Race/skin-color/ethnicity
DEC	Cross-section

#### Centrality and shortest paths

In order to analyse the influence of individual variables in the networks, we utilized the centrality function from the qgraph package, focusing on four specific centrality statistics. Closeness describes the extent to which a node has connections relative to its maximum possible in a given system, such that it can more easily “reach” many of variables. Strength reflects the relative weight of connections between a given node and its neighbors. Betweenness describes the number of shortest paths that pass-through a given node (i.e., the extent to which it binds the network together). Shortest paths represent the most efficient route between two variables, based on the absolute edge weights (relationship strengths). It allowed us to find the nodes that mediate the relationship between two variables, if there was no direct relationship present (Opsahl et al., 2010). Key variables are addressed in the Discussion.

#### Odds ratios

Whilst centrality statistics provided an impression of how variables work as part of the minority stress system, odds ratios were used to shift in the proportions of participants affected by the factors that affect suicide attempt. A summary of the variables that demonstrated significant changes in the odds of stressors or protective factors is shown in Table 3.

**Table 3:** **Odds ratios comparing item prevalence for NSS and SS groups in each cross-section**.

**Variable**	**2004–2006**			**2010–2012**		
	**SS (%)**	**NSS (%)**	**Ratio**	**SS (%)**	**NSS (%)**	**Ratio**
Sc6	77.0	84.1	0.63^*^	23.6	16.3	1.60^*^
NLE4	10.6	3.0	3.84^*^	1.6	2.1	0.66
NLE6	5.6	1.6	3.67^*^	2.0	0.7	2.62
NLE12	7.5	3.8	2.08^*^	2.6	1.1	2.59^*^
NLE13	8.1	4.7	1.77^*^	2.1	2.7	0.90
Pr1	54.4	30.3	2.74^*^	49.8	28.6	2.47^*^
Pr2	48.8	14.2	5.76^*^	88.5	8.0	87.16^*^
Pr4	47.6	17.3	4.32^*^	31.2	16.9	2.23^*^
Pr5	58.8	33.6	2.82^*^	46.5	24.0	2.75^*^
Pr6	44.7	14.1	4.92^*^	32.5	11.3	3.78^*^
Pr7	48.8	21.9	3.40^*^	41.5	16.8	3.50^*^
Pr8	46.0	21.4	3.13^*^	45.8	24.6	2.57^*^
Pr9	53.6	25.1	3.45^*^	54.0	32.6	2.42^*^
Pr10	27.2	3.8	9.43^*^	32.0	1.9	24.41^*^
Com3	6.8	2.7	2.60^*^	61.5	39.8	2.40^*^
Com4	7.4	2.8	2.77^*^	68.8	24.1	6.96^*^
Com5	3.9	1.1	3.67^*^	50.0	7.0	13.27^*^
Ag3	11.4	6.8	1.78^*^	83.6	72.7	1.93^*^
Ag4	7.4	3.4	2.26^*^	46.4	20.7	3.33^*^
Ag5	5.6	2.3	2.54^*^	15.0	5.2	3.19^*^
SAT	16.2	9.0	1.96^*^	25.4	7.2	4.42^*^

* *= < 0.01*.

*SS experience sexual stigma*.

*NSS no experience of sexual stigma*.

## Results

### Main outcomes

This study sought to compare changes in suicide attempt rates in young Brazilians between 2004 and 2012, focusing on differences between participants who had or had not experienced sexual prejudice. Whilst rates of suicide attempt decreased by 20% for those with no sexual stigma experiences (from 9.0 to 7.2%; Φ = 6.726, *p* < 0.01) between the two cohorts, rates of suicide attempt for those with experiences of sexual stigma increased by 60% (from 16.2 to 25.4%; Φ = 7.338, *p* < 0.01).

### Network analysis and centrality statistics

**Figure 1:**
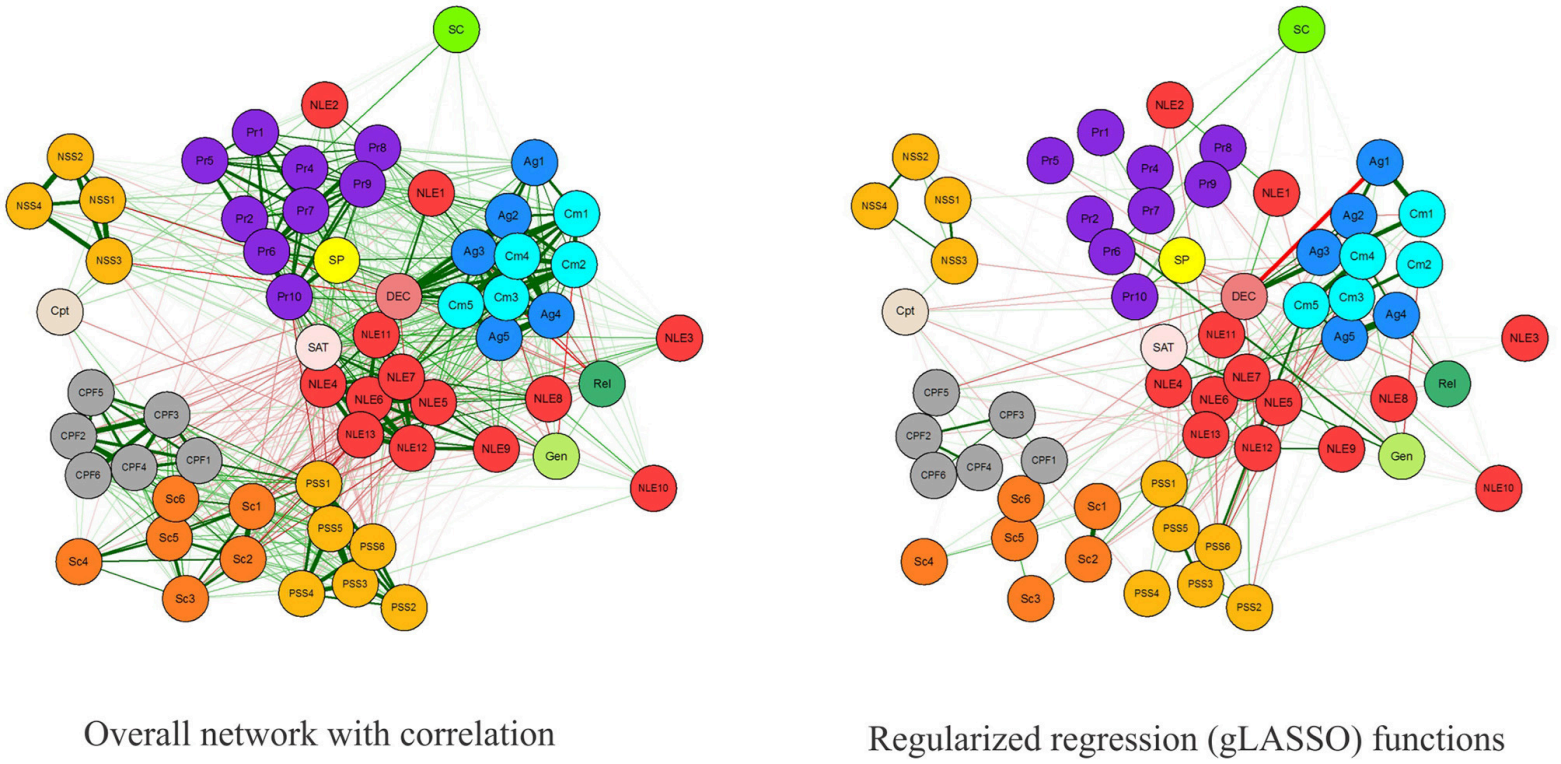
**Overall network with correlation (left)** and regularized regression (gLASSO) functions **(right)**. Variables names match those in Table 1.

**Figure 2:**
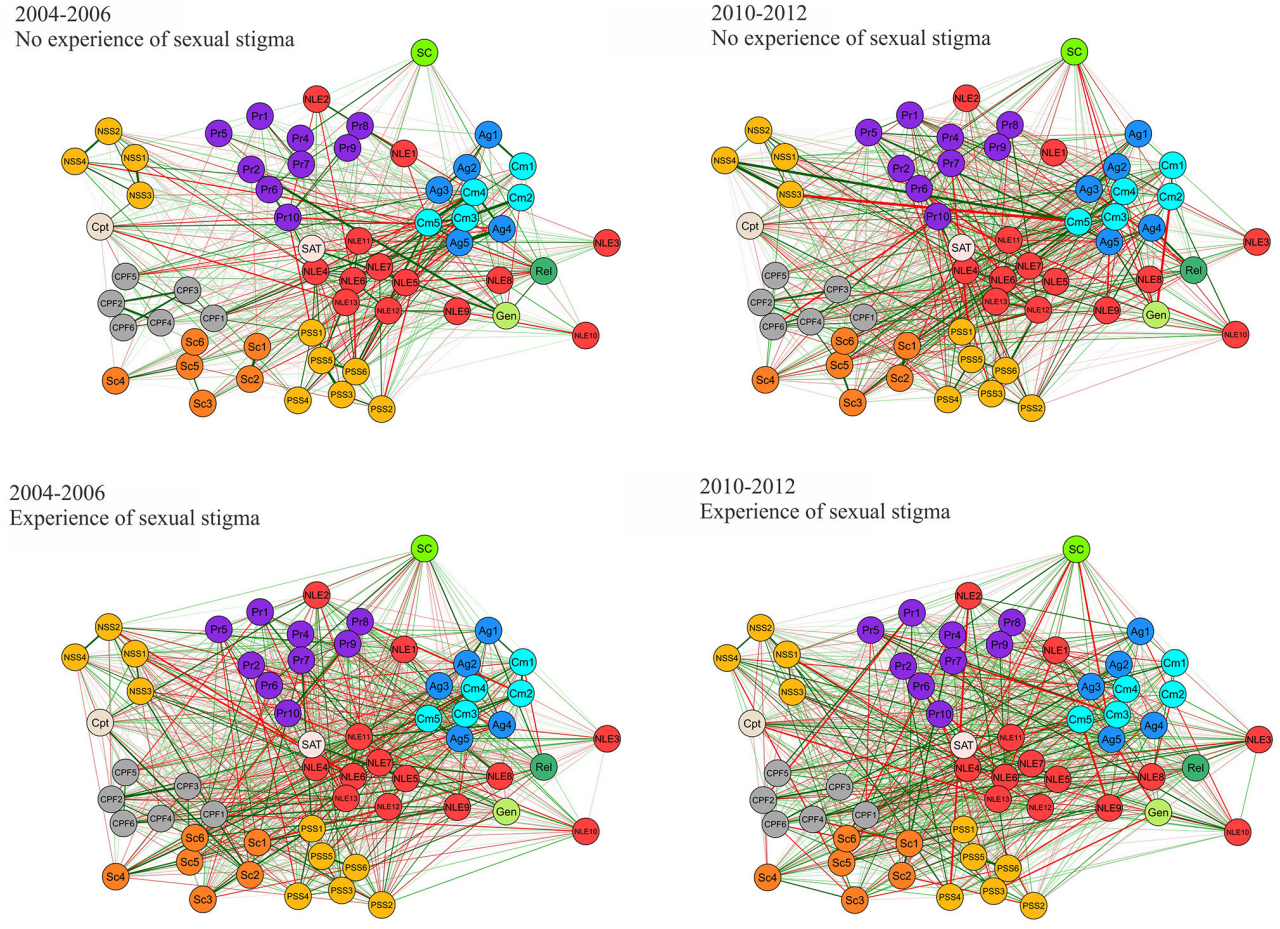
**gLASSO networks—experience of sexual stigma by cross-section**. Variables names match those in Table 1.

**Figure 3:**
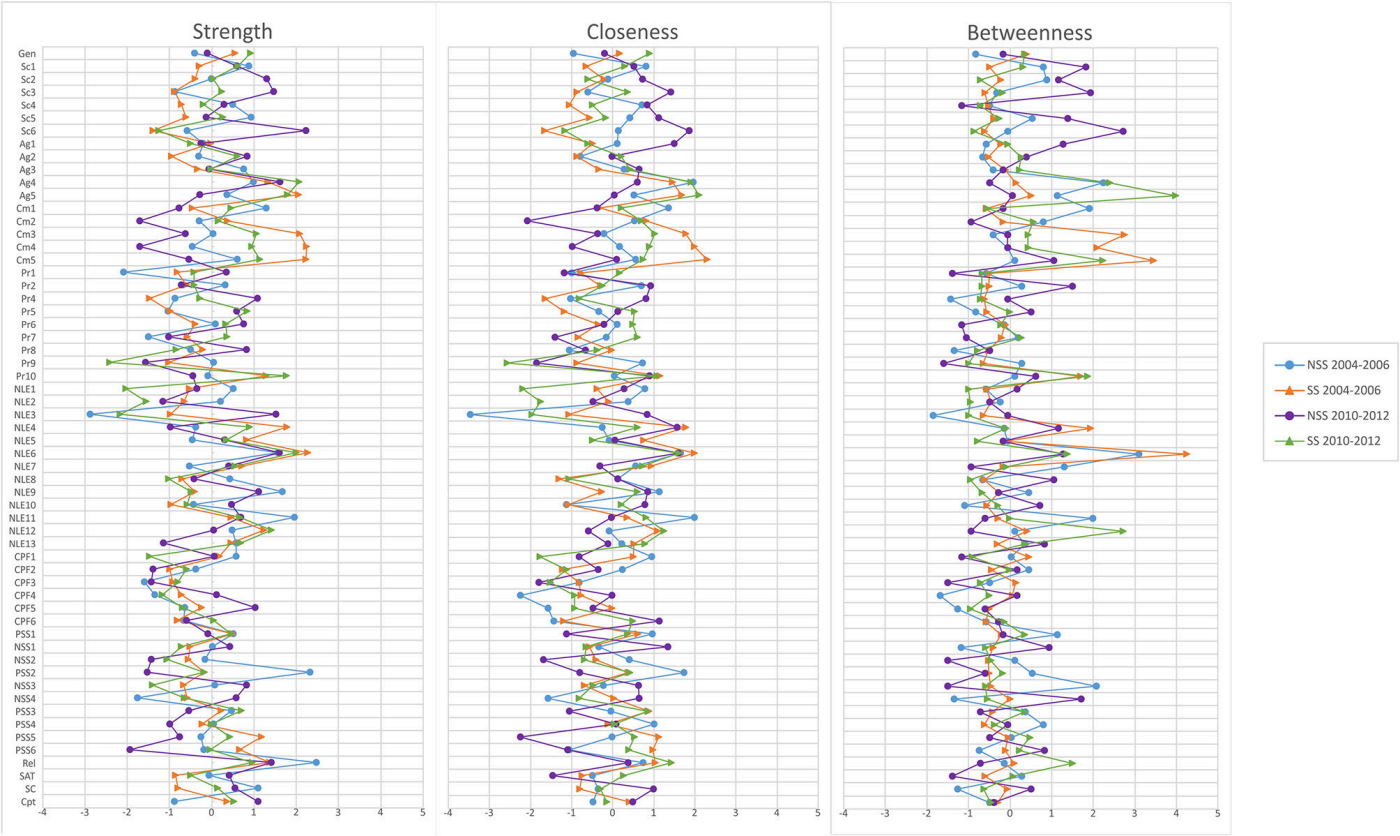
**Centrality statistics by cross-section and experience of sexual stigma**.

Network Analysis confirmed the existence of a Minority Stress Model in both cross-sections, with reliably clear divisions between protective and stress factors at a macro level. However, strong edges connecting several variables with the Decade variable in the Overall gLASSO graph (Figure 1) indicated that there have been significant shifts over time in a number of variables. The spatial structure from the Fruchterman-Reingold correlation graph layout has been maintained for comparative clarity. Furthermore, both the internal structure and changes across time in the most central variables within these graphs were distinctly different for those with sexual stigma experiences, in comparison with those who had not (Figure 2). These findings are also reflected in the centrality statistics. In both cross-sections, the variables with the strongest, closest, and greatest betweenness centrality are distinctly different for those with no experiences of sexual stigma vs. sexual stigma experiences (Figure 3); 1.6 mark the threshold for items with significantly high or low item betweenness, closeness, or strength. Items in the centrality analysis that are above 1.6 or below -1.6 may be interpreted as significantly high or low, respectively, in Betweenness, Closeness or Strength. Disparities between SS and NSS item centrality are key to this analysis, and are seen in the differences between markers. Furthermore, significant changes between cross-sections are marked by differences of 1.6 or greater. Key items are analyzed in the Discussion. Notably, the significant changes over time in variable centrality for these groups have almost no commonality (Figure 3). One variable that did remain constant for both groups was gender, demonstrating significantly more centrality in both cross-sections for those that have not experienced sexual stigma. Specifically, identifying as female was associated with greater odds of experiencing stressors in those that have not experienced sexual stigma, but play a non-significant role for those who experienced sexual stigma.

#### Shortest paths

Focusing on the independent and dependent variables, and the stressor variables that appear to have contributed most to systematic changes between the 2004–2006 and 2010–2012 cross-sections, sexual stigma, familial rape, familial molestation, and communal molestation were all directly related to suicide attempts. It is notable that, in the 2004–2006 cross-section, the relationship with communal rape was mediated by familial rape, perhaps alluding to ways in which a lack of familial support makes young people more vulnerable to communal abuse. However, in 2010–2012, all of these variables directly relate to suicide attempts, indicating that these environments had become more vulnerable for young people that had experienced sexual stigma. More details can be found in the Supplementary Material (Data Sheets 1–7).

#### Odds ratios

In terms of individual variable change, odds ratios for variables that experienced marked proportional change between 2004–2006 and 2010–2012 or between participants who experienced sexual stigma vs. those who did not are shown in Table 3.

## Discussion

Our results have demonstrated that, in the 2004–2006 cross-section, a sexual minority stress model, oriented around (perceived) sexual discrimination, existed amongst low-SES young Brazilians, undermining the well-being of those who had experienced sexual stigma. In this initial cross-section, these systems of inequality put young Brazilians who had experienced sexual stigma at 1.8 greater risk of attempting suicide than their counterparts. Moreover, the data reaffirm Meyer (2003) notion that minority experiences do not stand alone—they are markers for a wide array of disadvantage that tend to permeate an individual's life (Figure 1). Young people that have experienced sexual stigma were far more likely to also experience an array of negative life events, other kind of discrimination, and abuse from their family and/or community (Table 3). Moreover, low betweenness and closeness centrality statistics for suicide attempts in NSS participants (Figure 3) indicate that predictors of this outcome are more specific for those who had experienced sexual stigma. In practical terms, this means that certain stressors are likely to play a more central and severe role in the production of sexual minority suicide risk. However, it also means that it is possible to design more targeted responses to undermine the key stressors that comprise experience of sexual stigma.

The 2010–2012 cross-section demonstrated that suicide attempt rates for those who had not experienced sexual stigma had declined by 20%, whilst rates for their counterparts had increased by 60%. This is to say that, between 2004–2006 and 2010–2012, the odds ratio for suicide attempt, comparing those with and without experience of sexual stigma has more than doubled (from 1.96 to 4.42). Though just under 1 in 14 young people who had not faced sexual stigma attempted suicide (which is still higher than the national average—remembering this is a low-SES sample), over 1 in 4 attempted suicide amongst those who did face sexual discrimination (8.2 times former national estimates; Bertolote et al., 2005). Whilst there was a decline in rates of negative life events for all participants (albeit these were still disproportionately observed in the sexual minority group), we observed significantly greater increases in rates of all other forms of discrimination for sexual minority young people. One aspect were the increases in physical abuse with an object, molestation, and rape in both community and familial contexts. These increases occurred across the entire sample, however, the proliferation of these cases of abuse were at a much greater rate for those with experiences of sexual stigma in all cases, except familial abuse with an object (which was comparable). Whilst suicide attempts may not have been the most central to the sexual stigma experience network (Figure 3), these abuse variables represent a core risk in the sexual minority stress systems; a set of risks that have metastasized over the duration of this study.

It is plausible that the 2004–2006 cross-section may have under-reported these abuse statistics to some degree. Despite the production of the Brazilian Constitution (1988) and the Statute of the Child and Adolescent (1990), effective policy only began to be introduced in 2000, with the approval of the National Plan for Sexual Violence Against Children and Adolescents. Generational gaps in awareness could potentially explain the discrepancy in cognizance around these forms of abuse. This is supported by trends described by Safernet (http://www.childhood.org.br/numeros-da-causa): an increase in rates of reported abuse from 41,050 in 2004 to 189.211 in 2014. However, whilst rates of reported abuse in our study also increased overall, the disproportionately greater increases amongst those who had experienced sexual stigma suggest that increased awareness can only account for a fraction of this pattern. Furthermore, the notion that awareness generated by policy can account for this is undermined by the fact that there were no anti-abuse policies specifically aimed at LGBT populations, which suggests that the increases in reports of abuse amongst participants that experienced sexual stigma are even more noteworthy (i.e., that they are reporting more abuse despite the absence of awareness campaigns designed to target them). This reinforces the deduction that participants that experienced sexual stigma were not only worse off to begin with, but that their odds of encountering stressors are increasing at a greater rate than their counterparts with no experiences of sexual stigma.

Whilst the ratio between participants who experienced sexual stigma and those who did not regarding experiences of being beaten with an object by a community member decreased from 2.60 to 2.40, participants that have experienced sexual stigma were more likely than not to experience this form of abuse (61.5%). They also had almost a one in two chance to be molested by a family member (46.4%) and were even more likely to be molested by a community member (68.8%); at 2.24 and 2.85 times the rate of their counterparts. Most alarmingly, rates of familial rape almost tripled for those with experience of sexual stigma(2.88 times rates in participants without sexual stigma experience), and communal rape increased by almost 1300% (50.0%; 7.14 times rates in participants without sexual stigma experience). These systems of inequality are demonstrably worse. We consider it reasonable to conclude that this extreme level of abuse would lead to higher rates of suicide, due to the detrimental effect of such personal violation (Chang, 2015). This is corroborated by the 2004–2006 cross-section's shortest path statistics, which demonstrated that whether committed by a family or community member, molestation is linked to suicide attempt. This same can be said of familial rape. The relationship between suicide attempts and communal rape is mediated by familial molestation, which perhaps suggests that there are compounded vulnerabilities in these scenarios. However, by 2012, each one of these variables is directly linked to suicide attempts in the minority stress model.

Developing a rationale for these extreme patterns associated with experience of sexual stigma from within the data set, we looked at variables that showed similar patterns over the course of the study. Notably, there was a large spike in sexism (Pr2) experienced by young people with experiences of sexual stigma in 2010–2012. One explanation could be derived from Clausell and Fiske's (2005) study on homosexual stereotype content. This research demonstrated that the degree of homophobia espoused by participants was mediated by gender role conformity or, to use Lehavot and Lambert's (2007) term, gender expectancy violation. This has also been found in Brazil by Costa et al. (2012, 2015), however, these authors emphasize that heterosexism in Brazil aligns more with old-fashioned homonegativity (Herek, 1988), marked by moralistic objection to non-heterosexuality, rather than modern homonegativity more prevalent in Western nations (Morrison et al., 1999, 2009).

Glick et al. (2000) Ambivalent Sexism (for men and women) illustrated the manner in which gender role conformity is reinforced through “benevolent sexism,” whereby convergent behavior is privileged. Conversely, “hostile sexism” conceptualized the manner in which non-conformity is punished. During this period, conservatism in the Brazilian political sphere was on the rise (De Melo Modenesi et al., 2013). Following this line of reasoning, as sexism increased, leading to gender roles became more strictly policed, rates and severity of homophobic ideology and treatment would have followed. This is in line with the 22% increase in the proportion of young Brazilians reporting sexual discrimination. Similarly, sexist norms that privilege maleness may account for the disproportionate rate of female suicide attempts—just over three quarters of all attempts in both cross-sections.

One interesting discrepancy was that the role of gender in participants with experiences os sexual stigma was much less central than for those who hadn't experienced sexual stigma (Figure 3). In light of the role of gender expectancy violation, we suggest that the reason for the reduction of the effects of gender in participants who had experienced sexual stigma is because, rather than merely fitting into a patriarchal gender hierarchy of man over women, those who are perceived to be non-heterosexual are put into a subordinate third category of “gender violators.” This is congruent with Schope and Eliason's (2004) research on that also demonstrates how heterosexism is derived from gender role violation.

Experiences of sexual sitgma also significantly worsened over the period of the study, if not to the same degree. Subsequently, centrality statistics were employed to investigate whether other stress factors were compounding the vulnerabilities in young people who did not expericed sexual stigma. Figure 3 demonstrate some commonality between sexual stigma experience groups' centrality configurations in 2004–2006. However, by 2012 there are marked disparities across many of the variables and the role that they play in each groups' network. The elements that affect their risk for stress factors can be seen to have been transformed, presumably by changes in the social contexts that affect their lived realities. Simply put, there are structural differences in the experiences of young people with and without experiences of sexual stigma in low-SES Brazilian communities. Young people who have been subjected to sexual discrimination have fundamentally different experiences of the spaces that they share with those who have not had to survive such trauma. To reiterate, when we discuss sexual minority systems, we are discussing the entire milieu of stressor and protective factors that these young people are respectively more vulnerable to or denied because of their perceived sexuality. Whilst some factors are more central to these experiences than others, they constitute a significant part of the reality that renders suicide preferable to living in these situations.

### Sociopolitical context

In order to fully comprehend these heterosexist dynamics, one must understand the Brazilian political context around gender and sexuality issues. Over the course of this research, the Brazilian government has invested a great deal in policies that, at least at face value, promote diversity. These policies have aimed to ensure better access to higher education and affirm the identity of groups, such as afro-descendent, indigenous, and people with disability. Unfortunately, queer and trans communities have not received much attention under the umbrella of “diversity” (Moehlecke, 2009). In education, for example, the 2001 Brazilian National Education Plan failed to adequately address gender and sexuality education (Vianna and Unbehaum, 2004). The limited initiatives presented in this plan entailed courses for primary and secondary level teachers, taught in collaboration with university and NGOs; however, even those initiatives were short-lived (Mello et al., 2012). The most recent National Education Plan was approved in 2014, and all mention to gender or sexuality education was removed in response to pressure from (fundamentalist) religious members of congress, leaving Brazilian educational policy without any federal objectives regarding gender anti-discrimination for the next decade. So far, there has been little research to demonstrate the effects of this widespread political negligence or the impact of what little is being done. We believe the results of this study demonstrate indirectly the impact of the policy agenda that was partly implemented during former decades.

A key example of this line of intentional political neglect, and a major landmark in the eradication of gender and sexual educational content, took place in 2004. The Brazilian government instituted the *Brazil Without Homophobia* programme, which sought to target various systematic inequalities and issues in which non-heterosexual groups were over-represented, such as hate crime (Ramos and Carrara, 2006). Policy changes focused on securing civil and social rights that were previously exclusive to heterosexuals, and on targeting instances of discrimination within the legal system. Educational strategies included a sexual (and gender) equality campaign that was implemented through a series of booklets, posters, and videos, aimed at 6–9th grade students (in elementary and secondary school). Given the increasingly conservative political context, this move generated intense controversy. Pressure from fundamentalist religious lobbies (and their associated government representatives, whose power was growing) led President Dilma Rousseff to suspend these resources on the grounds that the material was apologetic (Brandão and Santana, 2011). The resources were never revised; schools were left without gender and sexuality resources; and the government demonstrated exactly where their priorities lay with regards to gender and sexual minority young people. We believe that it is not unreasonable to suggest that some degree of blame for the proliferation of violence toward individuals perceived to be non-heterosexual lies with the government's official stance toward these communities.

### Limitations

In this study we were unable to adequately explore whether there is systematic variation at the intersection of gender, sexual discrimination, skin color, and disability prejudice. Already, we have evidence that girls in this study conformed to the gender paradox around suicide attempts (representing over three quarters of those who attempted in both cross-sections; Canetto and Sakinofsky, 1998). We acknowledge that other research has demonstrated that skin color presents a multifaceted intersection with mental health in Brazil (Bastos et al., 2014). We have not assumed that young people's experiences are homogenous across these dimensions, but anticipate broader discussions of these intersections in future publications.

Significant differences were found for all demographic variables between cross-sections, however, we do not believe that these can account for the degree of change in sexual minority stress systems. In support of this, the gLASSO analyses, which partial out these differences, demonstrated that all demographic variables (bar gender) were peripheral in the networks, indicating they could not have influenced the model in this way. However, certain combinations of experience of sexual prejudice and these demographic variables may reveal more complex patterns of inequality. For example, skin color plays a more central role in experience of sexual stigma networks, suggesting that there may be a significant interaction. A detailed discussion of the effects of these differences is beyond the scope of this study, but we seek to discuss this in future publications oriented around the intersectional nature of this data.

## Conclusions

It would be unwise to suggest that any single factor could explain this increasingly heterosexist dynamic, nor that the key issues we have identified in this paper could account for all of the variance in this social degradation. However, it is important to highlight that conditions have worsened for those who face sexual discrimination. Their lived experiences have been rendered fundamentally different and more challenging as a result of these changes. In line with Dunn et al.'s (2014) Brazilian Sexual Minority Stress Model, it is patently obvious that young low-SES Brazilians who experienced sexual stigma are drastically more likely to face additional hardship, and are considerably less likely to have access to variables that may buffer the effects of these environments that have become increasingly dangerous for them throughout the duration of this research. The consequences of these circumstances are as simple as over one in four young people facing sexual discrimination having attempted suicide by 2010–2012. At almost four times the rate of their counterparts with no experiences of sexual stigma, and over double that again for national estimates, these conditions are an epidemic and require attention immediately.

## Author contributions

AB and SK designed the study. WM and AP conceptualized and ran the analysis. AB, SK, WM, AP, EA and LD wrote and approved the final version of the manuscript.

## Funding

This research was financed by the Excellence Research Center Support Program (PRONEX) of the Foundation for Research Support of the State of Rio Grande do Sul (FAPERGS), in partnership with the Brazilian National Council of Scientific and Technological Development (CNPq)

### Conflict of interest statement

The authors declare that the research was conducted in the absence of any commercial or financial relationships that could be construed as a potential conflict of interest.

## Supplementary material

Data Sheet 1 **Shortest Paths, experience of sexual stigma, 2004–2006: sph04.csv**.

Data Sheet 2 **Shortest Paths, no experience of sexual stigma, 2004–2006: spnh04.csv**.

Data Sheet 3 **Shortest Paths, experience of sexual stigma, 2012–2012: sph12.csv**.

Data Sheet 4 **Shortest Paths, no experience of sexual stigma, 2012–2012: spnh12.csv**.

Data Sheet 5 **Shortest Paths, total sample: SPpcortotal.csv**.

Data Sheet 6 **Partial correlation matrix: pcortotal.csv**.

Data Sheet 7 **Bivariate correlation matrix: cortotal.csv**.
